# Presenting features and treatment of small intestinal adenomyosis in children: a 10-year retrospective study

**DOI:** 10.3389/fped.2025.1555418

**Published:** 2025-03-24

**Authors:** Huan-sheng Wang, Ke-hua Huang, Ren-sen Jiang, Jin Lao, Jie-xing Long, Miao-bing Wu, Jia-lin Tang, Xian-ping Jiang, Bin Wang, Zi-min Chen, Jian-yao Wang

**Affiliations:** ^1^ Department of General Surgery, Shenzhen Children’s Hospital of China Medical University, Shenzhen, Guangdong, China; ^2^ Department of General Surgery, Shenzhen Children’s Hospital of ShanTou University, Shenzhen, Guangdong, China; ^3^ Department of General Surgery, Shenzhen Children’s Hospital, Shenzhen, Guangdong, China; ^4^ Department of Pathology, Shenzhen Children’s Hospital, Shenzhen, Guangdong, China

**Keywords:** acute abdomen, intussusception, intestinal obstruction, small intestinal adenomyomas, tumor

## Abstract

**Objective:**

This study aims to analyze and summarize the clinical characteristics of small intestinal adenomyosis.

**Methods:**

A retrospective study was conducted on children with small intestinal adenomyosis at our center from 2014 to 2024. The age of onset, gender, clinical symptoms, auxiliary examination results, treatment plans, and pathological characteristics of the tumors were recorded and analyzed.

**Results:**

Six cases of small intestinal adenomyosis were analyzed. The male-to-female ratio was 5:1. The median age was 19 months, with two-thirds of the affected children being under 2 years old. Two cases were identified in newborns during the treatment of intestinal malrotation. The remaining patients developed symptoms of intussusception. Preoperative ultrasound identified pathological lead points in two cases. Among the six cases, one case's adenomyoma was located in the jejunum, while the rest was located in the ileum, with tumors ranging from 25 to 140 cm from the ileocecal region. All six patients underwent resection of the tumor segments in the small intestine followed by anastomosis; postoperative prognosis was favorable. Of the six pathological results, glandular-like structures were found in five tumors, with three cases lined with cuboidal or columnar epithelium and one case lined with stratified or squamous epithelium.

**Conclusions:**

Small intestinal adenomyosis is observed to be more prevalent in male, with onset ages ranging from six days to four years. It frequently coexists with recurrent intussusception, making reduction with air or water enema challenging. Abdominal ultrasound typically shows tumors at the leading edge of the intussusception, with compromised blood flow signals and multiple small cystic or honeycomb-like anechoic structures. Surgery is the primary treatment and generally results in a favorable prognosis.

## 1 Introduction

Gastrointestinal adenomyosis, also known as myoepithelial hamartoma, adenomyomatous hamartoma, or anterior intestinal villous tumor, is a benign gastrointestinal mass with an unknown etiology. Previous literature suggests that adenomyosis mainly occurs in the pyloric region of the stomach, characterized by glandular structures lined with cuboidal to high columnar epithelium, surrounded by smooth muscle bundles (1, 2). Adenomyosis of the small intestine in children is exceedingly rare. According to literature review results, since Clarke et al. reported the first case in 1940, only 34 cases have been documented, with most being individual case reports (3–23). Due to the limited number of cases, the clinical characteristics of small intestinal adenomyosis in children remain unclear. Therefore, to increase awareness of this disease, we present the clinical characteristics, pathological features, auxiliary examinations, and treatment plans of children with small intestinal adenomyosis treated at our center over the past decade. Moreover, a comprehensive statistical analysis of adenomyosis cases reported both in the literature and the current study was performed, as detailed in the discussion section. This analysis encompassed sex-based incidence, age distribution, and the correlation between age at onset and tumor dimensions. To our knowledge, this study is the first to perform comprehensive analyses of the clinical characteristics of small intestinal adenomyosis in children.

## 2 Materials and methods

1. Study population: Cases with a final pathological diagnosis of adenomyoma of the small intestine, spanning from 2014 to 2024 at the Shenzhen Children's Hospital in Shenzhen, China, were collected. A total of six patients were enrolled, comprising five males and one female. Inclusion criteria: (1) postoperative pathological results confirmed as small intestinal adenomyoma; (2) complete medical records.
2. Clinical data: Clinical data collected in this study included age, sex, clinical symptoms, auxiliary examinations, surgical approach, tumor characteristics (location, size), and pathological findings from surgical specimens. Pathological findings were re-confirmed independently.
3. Statistical analysis: Patient demographics and clinical characteristics were reported using median and interquartile range (IQR). Linear regression and ANOVA were conducted using SPSS 24.0. Statistical significance was set at *p* < 0.05.

## 3 Results

### 3.1 General clinical information

In this study, two newborns were found to have tumors during the surgical treatment of intestinal malrotation, while the remaining children were diagnosed with intussusception prior to surgery. Among the six patients diagnosed with small intestinal adenomyosis, the ages ranged from 0.2 months to 56 months, with a median age of 19 months and an average age of 19.8 months. The male-to-female ratio was 5:1, with two-thirds of the patients being under two years old. Notably, there were two cases involving newborns: one was admitted for worsening jaundice and vomiting, and the other for repeated vomiting. The remaining patients presented with symptoms of intussusception, including abdominal pain, bloating, vomiting, and bloody stools, with vomiting being the most common symptom (Table 1).

**Table 1:** Clinical characteristics of patients with adenomyoma of the small intestine.

No	Age	Sex	Surgical diagnosis	Location	Size (cm)	Site	Distance to ileocecal valve (cm)	Distance to ligament of Treitz (cm)	Postoperative hospitalization days (day)	The followed-up result (>6 months)
1	6d	Male	Intestinal malrotation	Jejunum	0.8	NA	NA	10	20	Great
2	17d	Male	Intestinal malrotation	Ileum	1.0	NA	NA	NA	16	Great
3	1y4m	Female	Intussusception	Ileum	2.0	NA	25.0	NA	7	Great
4	2y1m	Male	Intussusception	Ileum	2.3	NA	35.0	NA	6	Great
5	1y9m	Male	Intussusception	Ileum	1.5	NA	30.0	NA	7	Great
6	4y8m	Male	Intussusception	Ileum	3.0	NA	140.0	NA	6	Great

d, days; m, months; NA, not available; y, years.

**Figure 1:**
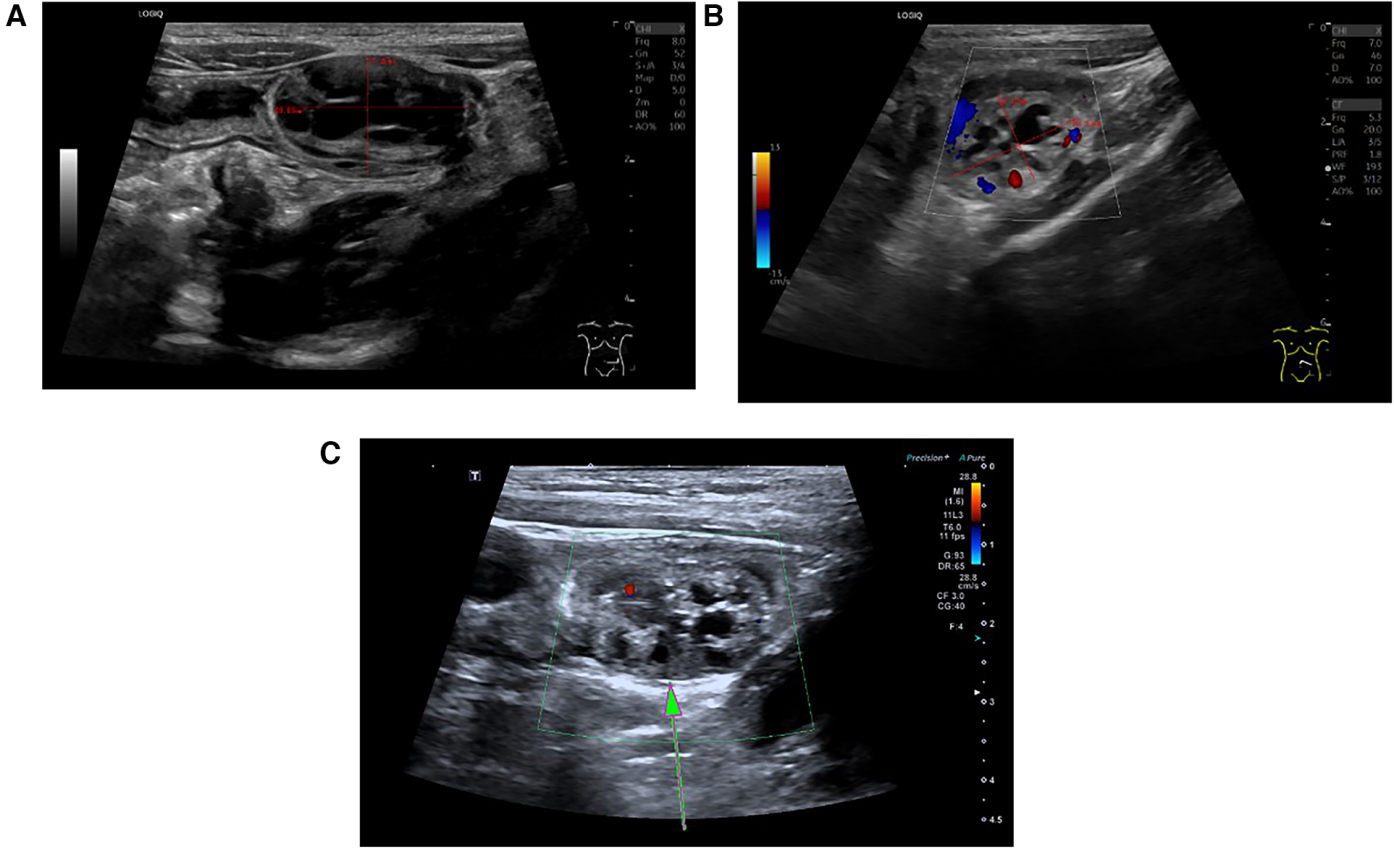
Classic ultrasound image of small intestinal adenomyoma. **(A)** On the ultrasound image, the small intestinal adenomyoma appears as a honeycomb-like structure, with a size of approximately 28.8 mm × 17.32 mm. **(B)** Small intestinal adenomyosis presents hypoechogenicity, with punctate or linear blood flow signals displayed within the mass. **(C)** The area pointed by the arrow is the small intestine adenomyoma.

All six patients underwent abdominal ultrasound examinations upon admission. The ultrasound results of four patients demonstrated the classic target ring sign in the short-axis view of the abdominal mass and the sleeve sign in the long-axis view, indicative of ileo-ileal type intussusception. The tumors were found at the head of the intussusception. The ultrasound features of the tumor included a hypoechoic mass at the head, with honeycomb-like or several small cystic echoes inside. Notably, the blood flow signals of the tumor were punctate, indicating poor blood supply (Figure 1). In case 5, the patient underwent abdominal CT enhancement and 3D reconstruction prior to surgery, but the CT results only revealed intussusception and did not detect any tumors in the small intestine.

### 3.2 Treatment and pathological results

**Figure 2:**
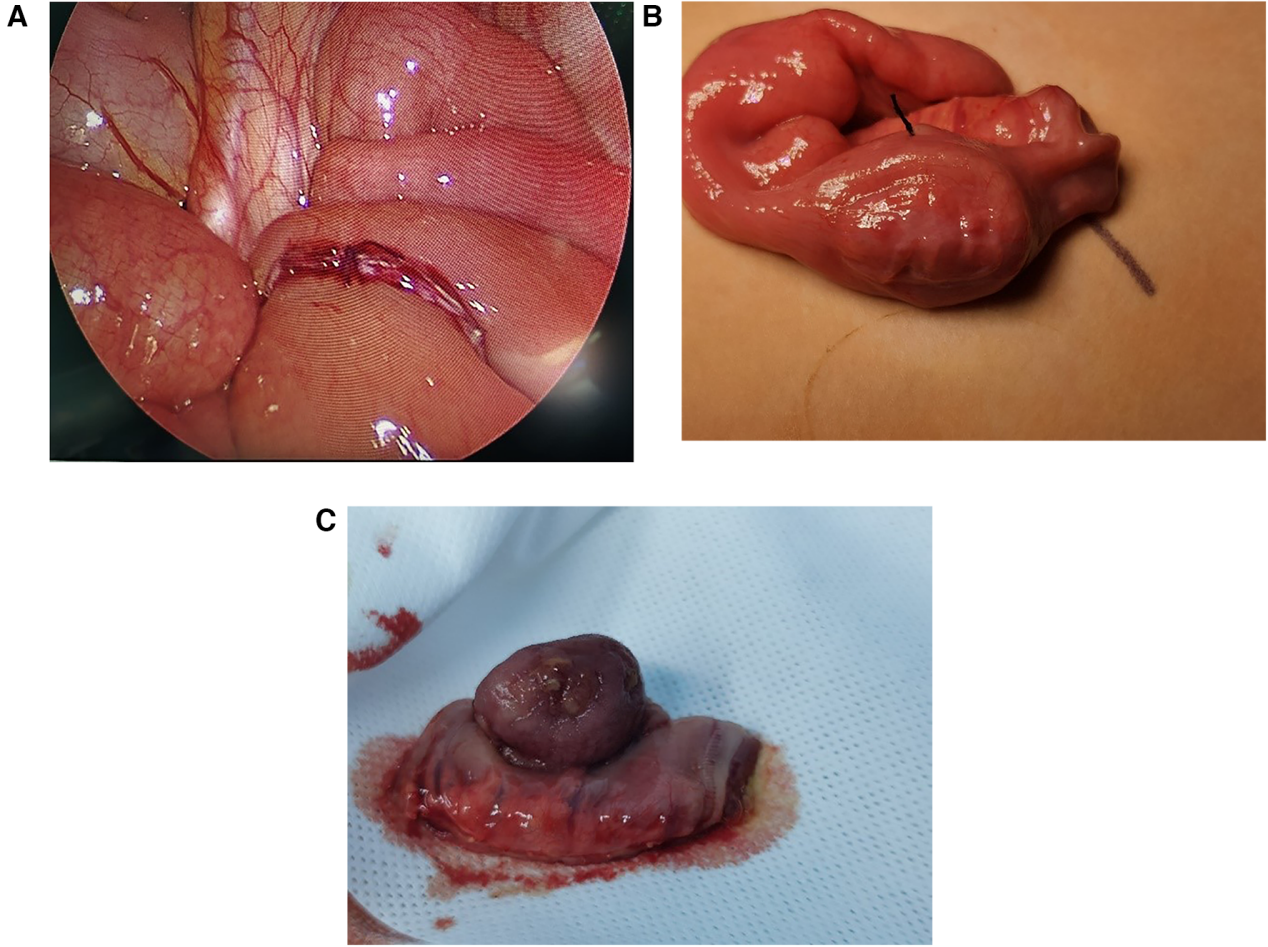
Intraoperative photos of small intestinal adenomyoma. **(A)** The ileum-ileum intussusception caused by small intestinal adenomyoma. **(B)** Small intestine segment containing small intestine adenomyoma. **(C)** The excised small intestinal adenomyoma, with 3 cm × 2 cm × 1 cm in size.

Among all the patients, one received ultrasound-guided water enema, which was not successful. All patients underwent laparoscopic surgery, which confirmed the presence of tumors in the small intestine during the operation. One of the patients had a tumor in the jejunum, located 10 cm from the duodenal suspensory ligament, while the others had tumors in the ileum, located 25–140 cm from the ileocecal region. Surgical management comprised complete tumor resection through small intestinal segmentectomy followed by immediate anastomotic reconstruction in each pediatric case. All six cases were initially explored using laparoscopy to locate the affected segment of the small intestine, which was then removed from the abdominal cavity for resection (Figure 2). In our study, intraoperative findings revealed that the tumors were located beneath the mucosa of the small intestine on the antimesenteric side. Quantitative assessment revealed that older children exhibited significantly larger resected tumor volumes compared to younger counterparts. Furthermore, no Meckel's diverticulum was identified within the intestine. The postoperative hospital stay ranged from 6 to 20 days, and the children's physical health was monitored during the six-month follow-up after discharge. Follow-up results indicated that the patients have remained free of tumor recurrence symptoms over the past six months (Table 1).

**Figure 3:**
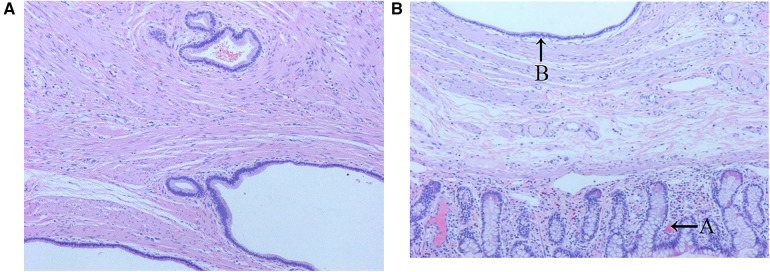
Classic pathological image of small intestinal adenomyoma (stained with hematoxylin and eosin, ×40). **(A)** Microscopic examination revealed that the tumor contains glandular structures of varying sizes interlaced with smooth muscle bundles. **(B)** Microscopic examination showed that arrow A points to the normal small intestinal mucosal epithelium, while arrow B arrow highlighted the glandular cavity with a dilated smooth muscle layer lined with a single layer of cuboidal epithelium.

Postoperative pathology revealed that all tumors were confined to the submucosal layer and covered by normal small intestinal mucosa. They protruded into the small intestinal lumen in a polyp-like manner, with diameters ranging from 0.8 to 3.0 cm. Histologically, all tumors exhibited glandular-like structures embedded within interlaced smooth muscles. Specifically, five of them were lined with cuboidal or columnar epithelium, while one was lined with stratified or squamous epithelium. No pancreatic-like tissue was detected in any of the tumor tissues (Figure 3).

## 4 Discussion

**Figure 4:**
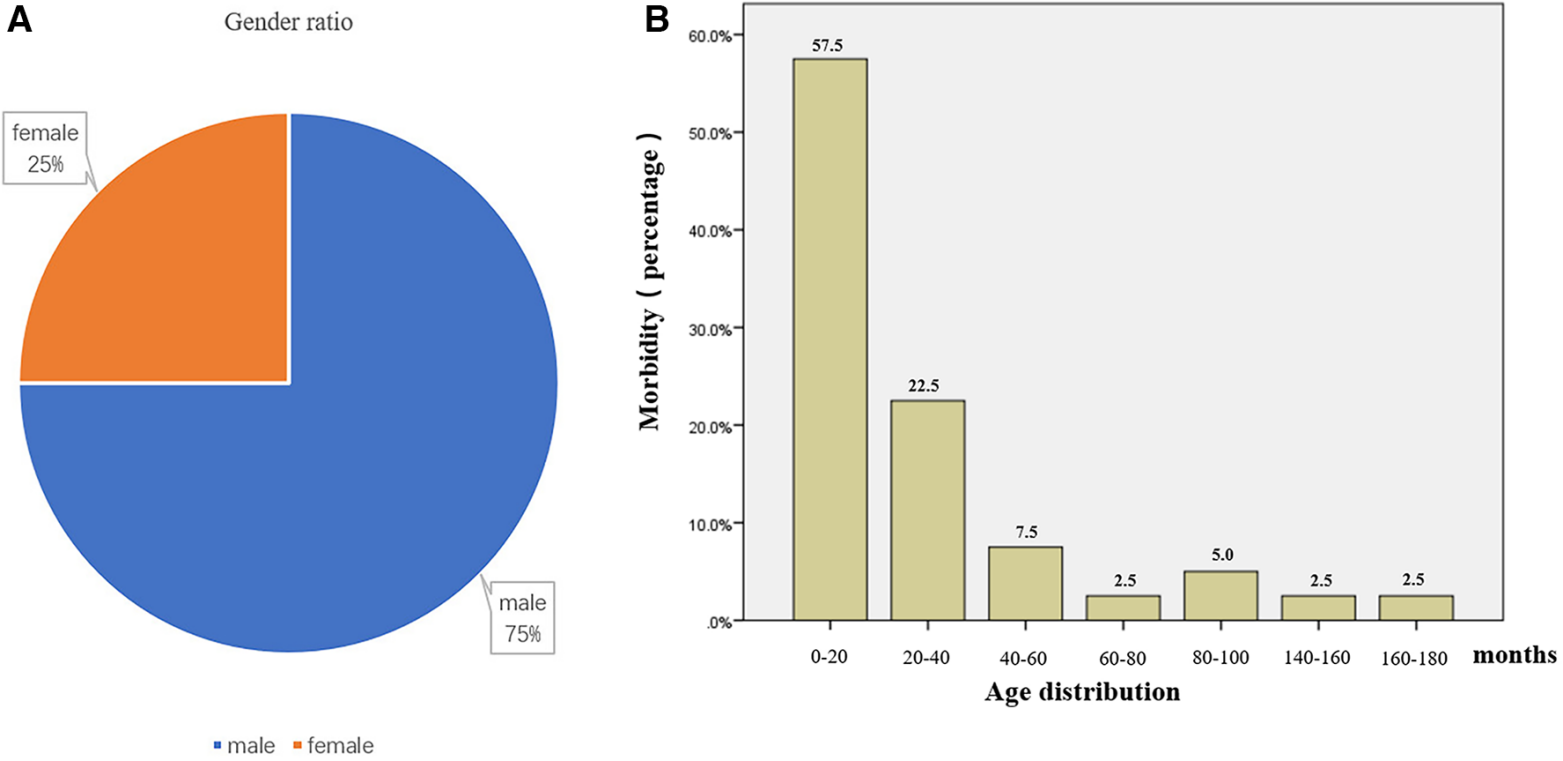
Gender and age distribution of patients with small intestinal adenomyosis. **(A)** According to statistics, the gender ratio of patients with small intestinal adenomyosis is 3:1 (male to female). **(B)** According to statistics, the age of onset of small intestinal adenomyosis in children is primarily concentrated between 0–20 months, comprising 57.5% of cases.

As a benign tumor-like lesion, adenomyosis can be observed in both adults and children and was first described by Magnus (24). In adult patients, adenomyoma was mostly found around the gallbladder and ampulla, with clinical symptoms typically including abdominal pain, jaundice, and fever (2, 25). In pediatric patients, the vast majority of adenomyosis are discovered due to recurrent symptoms of intussusception, such as repeated vomiting, abdominal pain, constipation, and feeding difficulties. Compared to adult patients, small intestinal adenomyosis in children is extremely rare and mostly reported as individual cases. To examine potential sex-based associations in small intestinal adenomyosis incidence, we performed comparative statistical analysis of both clinical variables (sex and disease occurrence). Based on literature reports and data from this study, adenomyosis of the small intestine is found to be more prevalent in male children, with a male-to-female ratio of 3:1. The age of onset is primarily concentrated between 0 and 20 months (Figure 4).

Currently, the pathogenesis of small intestinal adenomyosis is unclear. Some scholars believe that small intestinal adenomyosis should be considered a gastrointestinal hamartoma because the pathological tissue of the tumor contains goblet cells, silver protein cells, and smooth muscle matrix (8). This theory is supported by two additional case reports (14, 26). However, some scholars argued that this viewpoint lacks rigor, as the appearance of goblet cells and silver wax cells might be explained by metaplasia, while the presence of increased smooth muscle tissue could be due to secondary muscle proliferation caused by stimulation from displaced epithelium (27). Therefore, some scholars suggested that small intestinal adenomyosis might represent ectopic pancreatic tissue, based on the discovery of pancreatic acinar tissue in tumor (3). Babál et al. observed that the tissue chemical reactivity of duodenal adenomyosis in an elderly patient was similar to that of adjacent pancreatic ductal epithelium (28). Studies have reported that in the immunohistochemical findings of small intestinal adenomyoma, CK7 is positive and CK20 negative (26, 29). This finding paralleled the immunohistochemical results of the pancreatic duct epithelium, further supporting the theory of ectopic pancreas. In this research, no pancreatic tissue was detected in the postoperative pathology of any cases. However, various sizes of glandular structures and interlaced smooth muscle bundles around glandular elements were observed. The glandular lining consisted of non-atypical cuboidal, columnar, stratified, or squamous epithelium, aligning with the current pathological definitions of small intestinal adenomyoma (27). Therefore, additional studies are necessary to definitively characterize this condition.

In this study, two patients were found to have tumors during the surgical treatment for intestinal malrotation, while the others were diagnosed with intussusception prior to surgery, consistent with cases reported in the literature (Table 2). Intussusception is commonly diagnosed through abdominal ultrasound (30), which is internationally recognized as the preferred auxiliary examination due to its non-invasive, radiation-free, and readily available nature (31). Reports indicated that ultrasound examination for diagnosing intussusception has high sensitivity and specificity, and can sometimes detect small pathological lead points (32). We analyzed cases in the literature, among which twenty-three patients underwent ultrasound examination before surgery, and ten were found to have pathological lead points. Among the six cases in this study, four were diagnosed with intussusception by preoperative ultrasound examination, and three were found to have pathological lead points. In this study, the ultrasound features of small intestinal adenomyosis were as follows: (1) the small intestinal adenomyoma presents as a hypoechoic mass with uneven internal echoes and shows several small cystic/honeycomb shaped-structures with no echoes; (2) point/strip-shaped blood flow signals are displayed within the small intestinal adenomyoma. Notably, in case 5 of this study, the patient underwent both abdominal ultrasound and abdominal enhanced CT before surgery, but no pathological lead points were found on CT. According to literature reports, CT has difficulty identifying small intestinal adenomyosis when intussusception is present (7, 19). Therefore, we believe that ultrasound has higher sensitivity and specificity than CT in diagnosing small intestinal adenomyosis. Due to the small sample size, this comparison lacks persuasiveness, and more samples are needed for future research.

**Table 2:** Summary of case reports of children with adenomyoma of the small intestine in the literature.

No	First author	Year	Age	Sex	Surgical diagnosis	Location	Size (cm)	Site	Distance to ileocecal valve (cm)	Distance to ligament of Treitz (cm)
1.	Clarke et al. (21)	1940	15 y	Male	Intussusception	Meckel's diverticulum	NA	NA	NA	NA
2.	Schwartz et al. (5)	1958	8m	Male	Intussusception	Ileum	2.0	NA	25.0	NA
3.	Rosenmann et al. (6)	1980	2d	Female	Intussusception	Ileum	NA	NA	NA	NA
4.	Kim et al. (7)	1990	7y	Male	Intussusception	Ileum	4.0	Antimesenteric	NA	NA
5.	Gal et al. (8)	1991	9m	Male	Intussusception	Ileum	1.2	NA	70.0	NA
6.	Lamki et al. (9)	1993	22m	Male	Intussusception	Ileum	1.5	NA	NA	NA
7.	Serour et al. (10)	1994	3y9m	Male	Intussusception	Ileum	2.0	Antimesenteric	60.0	NA
8.	Chan et al. (11)	1994	5m	Female	Intussusception	Ileum	0.8	Antimesenteric	NA	NA
9.	Chan et al. (11)	1994	3y	Male	Incidental	Ileum	0.8	Antimesenteric	NA	NA
10.	Gonzalvez et al. (12)	1995	2y	Male	Intussusception	Ileum	2.0	NA	NA	NA
11.	Yamagami et al. (13)	2000	4m	Male	Intussusception	Ileum	NA	Antimesenteric	NA	NA
12.	Yao et al. (3)	2000	22m	Male	Intussusception	Meckel diverticulum	NA	NA	NA	NA
13.	Park et al. (14)	2003	7m	Male	Intussusception	Ileum	1.2	NA	20.0	NA
14.	Mouravas et al. (15)	2003	18m	Male	Intussusception	Ileum	1.5	NA	NA	NA
15.	Lo Bello Gemma et al. (23)	2003	13y	Female	Intussusception & volvulus	jejunum	NA	NA	NA	NA
16.	Ikegami et al. (16)	2006	5m	Female	Intussusception	Ileum	1.5	Antimesenteric	70.0	NA
17.	Bak et al. (17)	2014	11m	Female	intussusception	Ileum	1.0	NA	10.0	NA
18.	Copeland et al. (22)	2018	2m	Male	Gastroschisis	jejunum	1.5	NA	NA	NA
19.	Yan et al. (18)	2019	4m	Male	Intussusception	Ileum	1.0	NA	75.0	NA
20.	Yamada et al. (19)	2022	4m	Male	Intussusception	Ileum	1.8	NA	77.0	NA
21.	Blevrakis E et al. (20)	2023	2m	Female	intussusception	Ileum	NA	NA	NA	NA
22.	Li et al. (4)	2023	8y4 m	Male	Intussusception	Ileum	2.5	NA	260.0	NA
23.	Li et al. (4)	2023	1y4m	Male	Intussusception	Ileum	3.0	NA	40.0	NA
24.	Li et al. (4)	2023	2y6m	Female	Incidental	Ileum	1.5	Antimesenteric	150.0	NA
25.	Li et al. (4)	2023	7m	Male	Intussusception	Ileum	1.0	Antimesenteric	50.0	NA
26.	Li et al. (4)	2023	3y	Female	Intussusception	Ileum	3.0	Antimesenteric	30.0	NA
27.	Li et al. (4)	2023	1y	Male	Intussusception	Ileum	NA	Antimesenteric	30.0	NA
28.	Li et al. (4)	2023	11m	Male	Intussusception	Ileum	1.0	NA	30.0	NA
29.	Li et al. (4)	2023	2y4m	Male	Intussusception	Ileum	3.0	Antimesenteric	140.0	NA
30.	Li et al. (4)	2023	4m	Female	Intussusception	Ileum	1.5	Antimesenteric	110.0	NA
31.	Li et al. (4)	2023	3y10m	Male	Intussusception	Ileum	2.0	Antimesenteric	35.0	NA
32.	Li et al. (4)	2023	2m	Male	Intussusception	Ileum	1.0	NA	24.0	NA
33.	Li et al. (4)	2023	1m	Male	Intussusception	Ileum	NA	NA	30.0	NA
34.	Li et al. (4)	2023	6y7m	Male	Intussusception	Ileum	2.0	Antimesenteric	75.0	NA

d, days; m, months; NA, not available; y, years; cm: centimeter.

**Figure 5:**
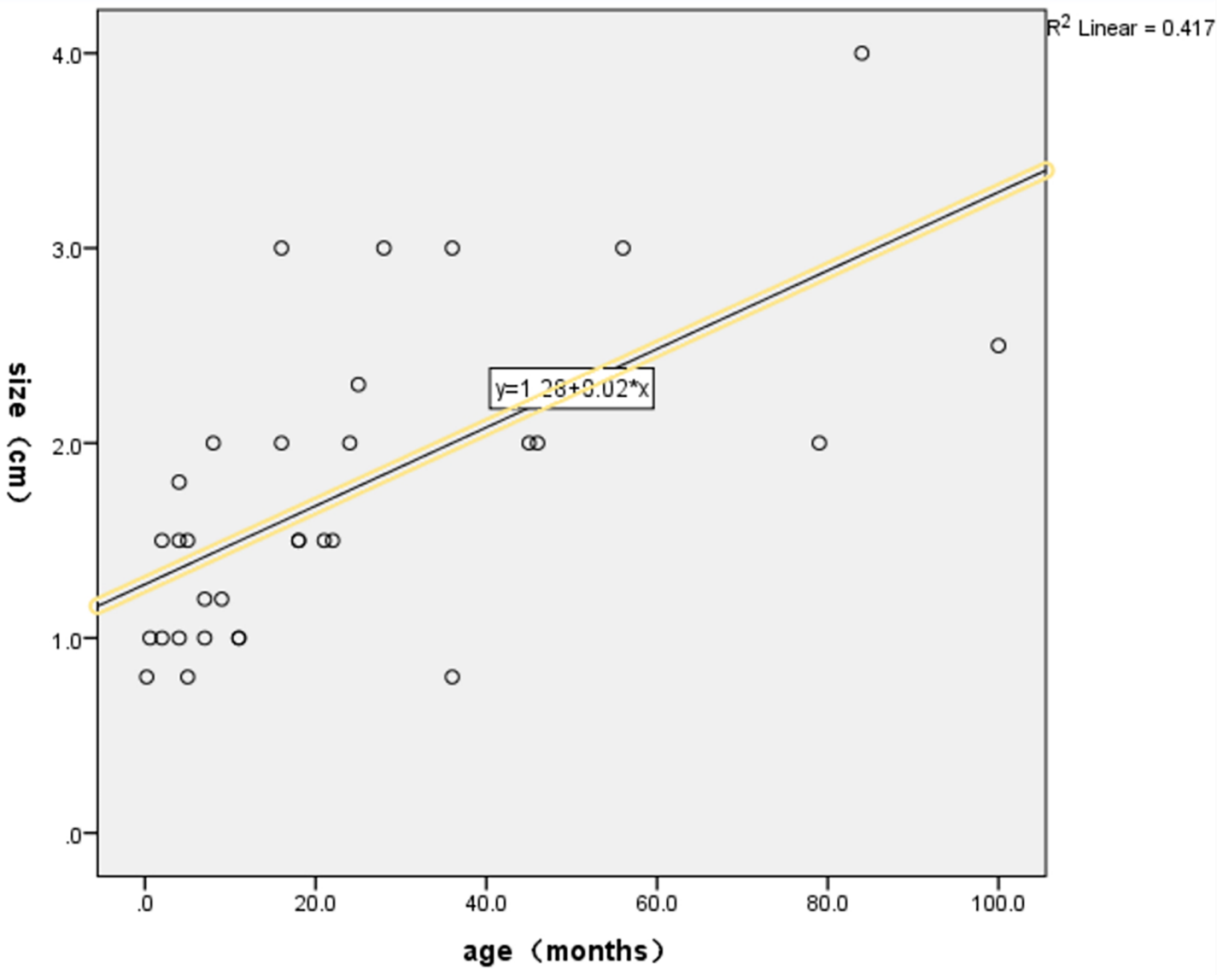
Statistical relationship between tumor size and age of onset. The scatter plot demonstrated a positive linear relationship between tumor size and age of onset, and a corresponding linear regression equation was derived. SPSS analysis results showed that the linear regression equation between tumor size and age of onset was statistically significant (*p* < 0.05).

Because most children with small intestinal adenomyosis often present with acute abdomen upon admission, the majority receive emergency surgical treatment. Among the six cases in this study, one child underwent ultrasound-guided water enema treatment before surgery, but the intussusception could not be released. Our systematic review of existing literature revealed that neither pneumatic nor hydrostatic enema procedures demonstrated successful reduction of intussusception in any preoperative clinical documentation (4, 13, 16, 17, 19, 20). This suggested that the use of air or water enema to reduce intussusception caused by small intestinal adenomyosis presents significant challenges. Therefore, all six patients underwent small intestine segment resection and end-to-end anastomosis at the tumor site. This surgical procedure is advantageous due to minimal trauma, a clear surgical field, and short surgical time, and is recommended in the literature (4). The scope of resection for diseased intestinal segments is rarely mentioned in the literature. We recommend resecting the affected intestinal segment based on findings from preoperative auxiliary examinations and intraoperative assessments. If the affected segment was non-necrotic, resection should be performed 2 cm distal to the tumor, followed by intestine-to-intestine anastomosis. In cases of necrosis, the affected intestine segment should be resected in accordance with the extent of the necrotic tissue. Finally, if additional lesions are identified during surgery, such as congenital intestinal malrotation, Meckel's diverticulum, or small intestinal torsion, the corresponding intestinal segment should be removed. In this study, except for case 1 where the tumor was located in the jejunum, all other tumors were located in the ileum. A literature review found that only two cases had tumors located in the jejunum (22, 23), while the others were located in the ileum. This indicates that the majority of small intestinal adenomyosis occur in the ileum. Additionally, an analysis of the relationship between tumor size and age of onset, based on case reports and our data, revealed a linear correlation between the two variables. Specifically, a positive correlation was observed between tumor size and age of onset (Figure 5).

In summary, adenomyoma of the small intestine in children is exceedingly rare. Although it is a benign tumor, its etiology remains unclear. It frequently coexists with recurrent intussusception, making reduction with air or water enema challenging. Abdominal ultrasound typically shows tumors at the leading edge of the intussusception, with compromised blood flow signals and multiple small cystic or honeycomb-like anechoic structures within. Surgery is the primary treatment and generally results in a favorable prognosis.

Given the retrospective nature of this study, selection bias may be present. Additionally, the study primarily focuses on perioperative complications, and the follow-up duration is relatively brief. All patients included in the study originated from a single center, which may not be representative of the entire population.

## Data Availability

The original contributions presented in the study are included in the article/Supplementary Material, further inquiries can be directed to the corresponding author.
